# A decagonal quasicrystal with rhombic and hexagonal tiles decorated with icosahedral structural units

**DOI:** 10.1107/S2052252520004297

**Published:** 2020-04-23

**Authors:** W. Z. Wang, X. Z. Zhou, Z. Q. Yang, Y. Qi, H. Q. Ye

**Affiliations:** a School of Materials Science and Engineering, Northeastern University, Shenyang 110819, People’s Republic of China; b Shenyang National Laboratory for Materials Science, Institute of Metal Research, Chinese Academy of Science, Shenyang 110016, People’s Republic of China

**Keywords:** decagonal quasicrystals, icosa­hedrons, Penrose tiling, electron diffraction, scanning-transmission electron microscopy

## Abstract

The structure of a decagonal quasicrystal in the Zn_58_Mg_40_Y_2_ (at.%) alloy was studied using electron diffraction and atomic resolution *Z*-contrast imaging techniques. This stable Frank–Kasper Zn–Mg–Y decagonal quasicrystal has an atomic structure which can be modeled with a rhombic/hexagonal tiling decorated with icosahedral units at each vertex. No perfect decagonal clusters were observed in the Zn–Mg–Y decagonal quasicrystal, which differs from the Zn–Mg–Dy decagonal crystal with the same space group *P*10/*mmm*. Y atoms occupy the center of ‘dented decagon’ motifs consisting of three fat rhombic and two flattened hexagonal tiles. About 75% of fat rhombic tiles are arranged in groups of five forming star motifs, while the others connect with each other in a ‘zigzag’ configuration. This decagonal quasicrystal has a composition of Zn_68.3_Mg_29.1_Y_2.6_ (at.%) with a valence electron concentration (*e*/*a*) of about 2.03, which is in accord with the Hume–Rothery criterion for the formation of the Zn-based quasicrystal phase (*e*/*a* = 2.0–2.15).

## Abstract 2

A new kind of phasonic entropy-stabilized decagonal quasicrystal (space group *P*10/*mmm*) was observed in the Zn–Mg–Y system, in addition to an icosahedral quasicrystal and a decagonal quasicrystal (space group *P*105/*mmc*) which have been reported previously. This decagonal quasicrystal can be modeled with dense packing of icosahedrons at vertices of fat rhombic and flattened hexagonal tiles.

## 1. Introduction   

Quasicrystals are long-range-ordered solids with icosahedral, decagonal and other non-crystallographic symmetries (Shechtman *et al.*, 1984 ▸; Wang *et al.*, 1987 ▸; Yamamoto, 1996 ▸; Takakura *et al.*, 2007 ▸; Subramanian *et al.*, 2016 ▸; He *et al.*, 2016 ▸). To date, a large number of quasicrystals, including three-, two- and one-dimensional quasicrystals, have been found in various materials such as alloys, oxides and soft materials (Wang *et al.*, 1987 ▸; Tang *et al.*, 1993 ▸; Abe *et al.*, 1999 ▸; Hiraga *et al.*, 2001 ▸, 2015 ▸; Hayashida *et al.*, 2007 ▸; Bindi *et al.*, 2012 ▸; Förster *et al.*, 2013 ▸). Quasicrystals in alloys are traditionally categorized into two groups based on their chemistry and local order: Al transition-metal (Al-TM) and Frank–Kasper (F-K) classes. Decagonal quasicrystals existing in both groups are one type of two-dimensional quasicrystals, with a tenfold axis (*c* axis) and periodicity along this axis (Bendersky, 1985 ▸; Burkov, 1991 ▸; Hiraga *et al.*, 1991 ▸, 1993 ▸, 1994 ▸; Sato *et al.*, 1997 ▸; Abe & Sato, 1998 ▸; Abe *et al.*, 1999 ▸). The diffraction pattern recorded parallel to the *c* axis is similar to that of Penrose tiling, which suggests that projection of the decagonal quasicrystal along this axis is closely related to the structure of Penrose tiling (Ishihara & Yamamoto, 1988 ▸; Yamamoto & Ishihara, 1988 ▸; Levine & Steinhardt, 1986 ▸; Yamamoto, 1996 ▸). Intensive high-resolution transmission election microscopy (HRTEM) investigations highlight that these structures can be described as periodic packing of various ‘quasi-unit-cells’, which are located at the vertices of the Penrose tiles (Steurer & Kuo, 1990 ▸; Hiraga *et al.*, 1994 ▸; Gummelt, 1996 ▸; Steinhardt *et al.*, 1998 ▸; Abe *et al.*, 2004 ▸; Yasuhara & Hiraga, 2015 ▸). These quasi-unit-cells are known to consist of atomic columns, existing in the corresponding approximant crystalline phases. So the atomic structure of decagonal quasicrystals can be deduced from their crystalline approximants.

For Al-TM decagonal quasicrystals, their structure, physical properties, thermodynamic stability and chemical bonding have long been investigated (Steurer & Kuo, 1990 ▸; Hiraga *et al.*, 1991 ▸, 1993 ▸, 1994 ▸; Abe *et al.*, 2003 ▸). Icosahedral clusters are ubiquitous in Mg–Zn Laves phases (such as MgZn_2_ and Mg_4_Zn_7_), and juxtaposed packing of icosahedral chains is a characteristic feature of F-K intermetallic compounds (Frank & Kasper, 1958 ▸; Wang *et al.*, 1986 ▸; Ye *et al.*, 1985 ▸; Kuo *et al.*, 1986 ▸; Yang *et al.*, 2018 ▸; Zhang *et al.*, 2018 ▸). However, studies on the F-K class of decagonal quasicrystals have far fewer outputs than the Al-TM group so far. A kind of decagonal quasicrystal (space group *P*10_5_/*mmc*) with overlap tiling of quasi-unit-cells about 2.3 nm in diameter was observed in Zn–Mg–RE (where RE is a rare-earth element) alloys (Abe & Sato, 1998 ▸; Abe *et al.*, 1999 ▸). A 2.3 nm quasi-unit-cell was constructed based on the atomic structure of F-K compound Mg_4_Zn_7_ (Abe *et al.*, 1999 ▸). Single-crystal X-ray diffraction and atomic resolution high-angle annular dark-field scanning transmission electron microscopy (HAADF-STEM) imaging found an F-K-type Zn–Mg–Dy decagonal quasicrystal consisting of decagonal clusters (∼2.3 nm in diameter) on a pentagon–Penrose tiling and star-like clusters (∼2.4 nm in diameter) covering the remaining space, and there are icosahedral units in both kinds of clusters (Ors *et al.*, 2014 ▸). Decagonal clusters are allowed to overlap in the Zn–Mg–Dy decagonal quasicrystal, but no icosahedral units exist at their centers (Ors *et al.*, 2014 ▸). In addition, three-dimensional icosahedral quasicrystals have been widely observed in various Mg–Zn–RE alloys (Luo *et al.*, 1993 ▸; Liu *et al.*, 2015*a* ▸,*b* ▸). Furthermore, Monte Carlo and molecular dynamics simulations predicted the existence of a decagonal quasicrystal which can be modeled with a rhombic/hexagonal tiling decorated with icosahedral clusters in Mg–Zn–RE alloys (Mihalkovič *et al.*, 2014 ▸). Simulations found that all fat rhombi were arranged in groups of five, forming star motifs (Mihalkovič *et al.*, 2014 ▸). However, there is no experimental verification of the formation of such predicted decagonal quasicrystal in Mg–Zn–RE alloys.

In this paper, we report a decagonal F-K quasicrystal with simple icosahedrons consisting of 13 atoms as the building units in a Zn_58_Mg_40_Y_2_ (at.%) alloy. The space group and atomic occupation of the ternary quasicrystal were determined based on electron diffraction and atomic resolution *Z*-contrast imaging investigations. The structure units of this decagonal quasicrystal are simple icosahedrons which are much smaller than those in Al-TM and Zn–Mg–Dy decagonal quasicrystals (Abe & Sato, 1998 ▸; Abe *et al.*, 1999 ▸; Ors *et al.*, 2014 ▸). The present Zn–Mg–Y decagonal quasicrystal has the same space group (*P*10/*mmm*) as the Zn–Mg–Dy decagonal quasicrystal (Ors *et al.*, 2014 ▸), but shows different atomic structures. In addition, a high fraction of fat rhombi are arranged to form configurations other than star motifs in the present Zn–Mg–Y decagonal quasicrystal, which is thus somewhat different from the model predicted by simulations (Mihalkovič *et al.*, 2014 ▸).

## 2. Experimental details   

A Zn_58_Mg_40_Y_2_ (at.%) alloy was produced by high-frequency induction melting under an argon atmosphere. Samples cut from the ingot were annealed at 673 K for 100 h, then quenched into water. Specimens for transmission electron microscopy investigations were prepared using standard ion-milling techniques. Tilt series of selected area electron diffraction (SAED) patterns were obtained using a Tecnai G^2^ F30 microscope. Convergent-beam electron diffraction (CBED) patterns were taken on a JEOL 2100 microscope. Atomic resolution HAADF-STEM observations and atomic resolution energy dispersive X-ray (EDX) mapping were carried out on an aberration-corrected Titan 60–300 microscope equipped with a super EDX spectroscopy detector.

## 3. Results and discussion   

**Figure 1:**
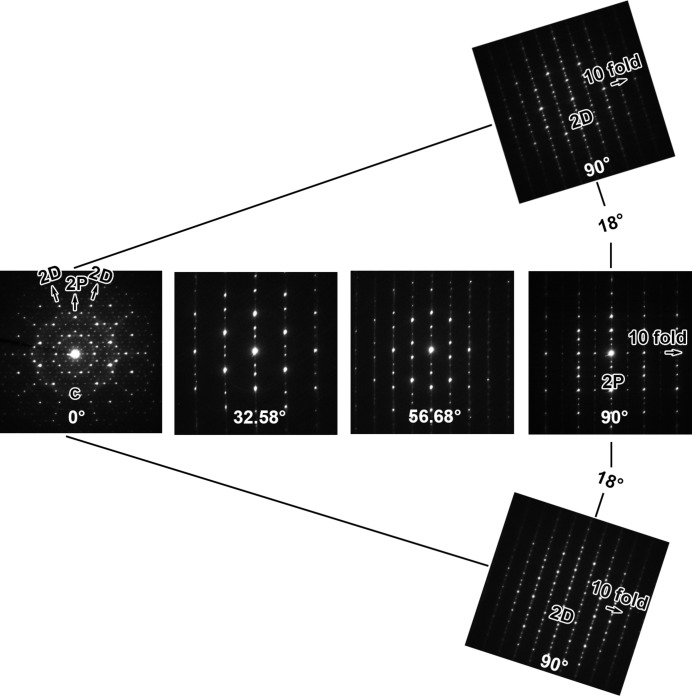
Tilt series of SAED patterns of the decagonal quasicrystal phase.

Fig. 1 ▸ presents a tilt series of SAED patterns obtained from Zn–Mg–Y quasicrystals in annealed samples, showing clear characteristics of decagonal symmetry (Hiraga *et al.*, 1993 ▸). A large number of sharp diffraction spots at tenfold symmetry positions can be seen from the SAED pattern along the *c* axis, indicating clearly on-average long-range quasi-periodic order in this decagonal quasicrystal. The SAED patterns obtained along twofold axes, designated as the 2D and 2P directions, show a regular periodicity of 0.51 nm along the tenfold direction, which is the same as the periodicity of the Zn–Mg–Dy decagonal quasicrystal along the tenfold axis (Abe & Sato, 1998 ▸). However, no reflections along the tenfold axis disappeared during tilting of the specimen from the 2D to the 2P direction, in contrast to that of the Zn–Mg–Dy decagonal quasicrystal with the space group *P*10_5_/*mmc* (Abe & Sato, 1998 ▸; Abe *et al.*, 1999 ▸).

**Figure 2:**
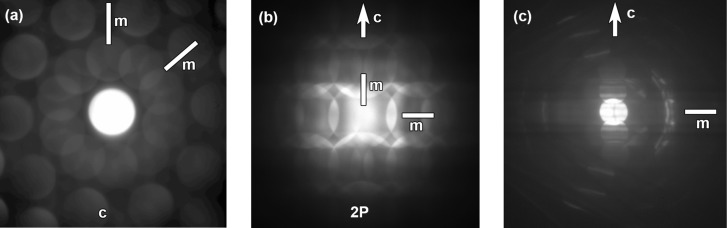
CBED patterns obtained along (*a*) the *c* axis, (*b*) 2P direction and (*c*) a direction perpendicular to the *c* axis.

In order to reveal the space group of the Zn–Mg–Y decagonal quasicrystal, CBED patterns were taken along the *c* axis [Fig. 2 ▸(*a*)] and the directions perpendicular to the *c* axis [Figs. 2 ▸(*b*) and 2(*c*)]. Fig. 2 ▸(*a*) shows a CBED pattern with a tenfold rotation symmetry around the *c* axis and two sets of mirror symmetries parallel to the *c* axis, *i.e.* a 10*mm* symmetry. The CBED pattern recorded along the 2P direction reveals mirror symmetries both parallel and perpendicular to the *c* axis, as shown in Fig. 2 ▸(*b*). Since CBED patterns shown in Figs. 2 ▸(*a*) and 2(*b*) are formed only by zeroth-order Laue zone reflections, a twofold axis perpendicular to the incident beam acts equivalently to a mirror plane parallel to the incident beam (Buxton *et al.*, 1976 ▸). Two possible point groups, 10/*mmm* and 10/222, can be deduced for this Zn–Mg–Y decagonal quasicrystal. A CBED pattern including high-order Laue zone (HOLZ) reflections should be obtained in order to determine the point group exclusively. Fig. 2 ▸(*c*) shows a CBED pattern containing HOLZ reflections taken along the direction perpendicular to the *c* axis, but that deviated from the 2P direction. This CBED pattern shows a mirror symmetry perpendicular to the *c* axis, which means that the Zn–Mg–Y decagonal quasicrystal has a centrosymmetric point group. The point group of the decagonal quasicrystal is thus determined to be 10/*mmm*. In addition, there are no reflections due to presence of a superlattice structure and no extinction of diffraction as a result of the presence of a 10_5_ screw axis and *c*-glide plane. Therefore, the space group of this Zn–Mg–Y decagonal quasicrystal should be *P*10/*mmm*, which is consistent with the result of the Zn–Mg–Dy decagonal qusicrystal reported by Örs *et al.* (2014 ▸). In order to reveal the atomic structure of the present Zn–Mg–Y decagonal quasicrystal and its relationship with the previously reported Zn–Mg–Dy decagonal quasicrystal (Ors *et al.*, 2014 ▸), atomic resolution HAADF-STEM imaging was carried out along both the tenfold and twofold zone axes.

**Figure 3:**
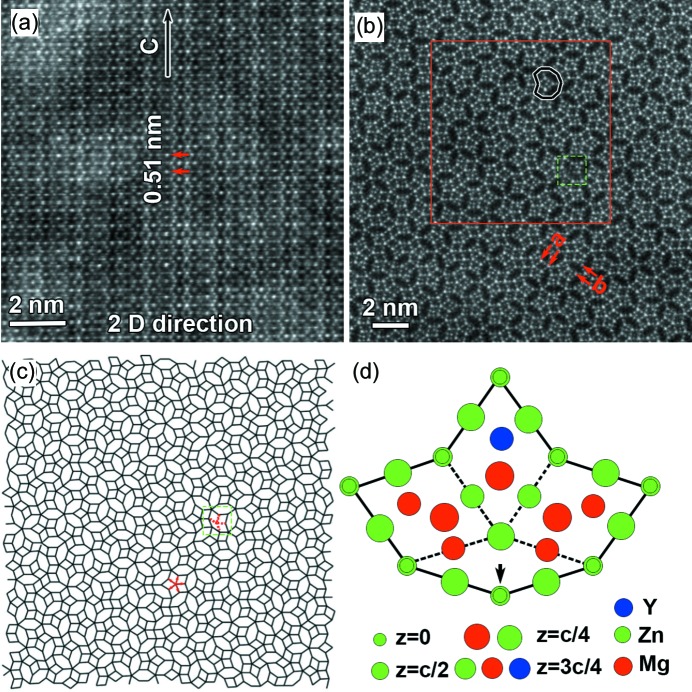
Atomic resolution HAADF-STEM images taken along (*a*) the 2D direction and (*b*) tenfold axis. (*c*) Schematic of the tiling for the region shown in (*b*). (*d*) Atomic structure model for the boat tile indicated in (*b*) and (*c*).

**Figure 4:**
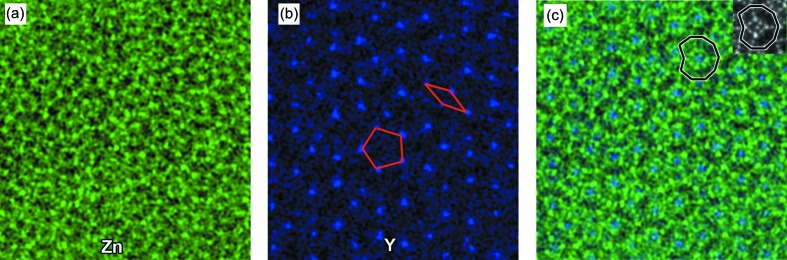
Elemental maps of (*a*) Zn and (*b*) Y, corresponding to the area outlined by the red square in Fig. 3 ▸(*b*). (*c*) Complex elemental maps of Zn and Y in (*a*) and (*b*), respectively. Inset in (*c*) is an atomic resolution HAADF-STEM image of the ‘dented decagon’ motif.

Figs. 3 ▸(*a*) and 3(*b*) present atomic resolution HAADF-STEM images obtained along the 2D and tenfold directions, respectively. Periodicity along the tenfold axis and quasi-periodicity perpendicular to this axis can be seen clearly from the image recorded along the 2D direction, as shown in Fig. 3 ▸(*a*). When viewed along the tenfold axis, strong bright image spots are arranged into fat rhombic and flattened hexagonal tiles which are distributed aperiodically, as shown in Figs. 3 ▸(*b*) and 3(*c*). Edges of both kinds of tiles have the same length of about 0.45 nm. The intensities of image spots located at the vertices are about twice those at the mid-edges. These characteristic parameters of fat rhombic and flattened hexagonal tiles in the present Zn–Mg–Y quasicrystal are as the same as those in the F-K Mg_4_Zn_7_ crystal (Abe *et al.*, 1999 ▸; Yang *et al.*, 2018 ▸). These brighter dots at the vertices correspond to central Zn atomic columns of icosahedral chains projected along their pseudo-fivefold axis in F-K compound crystals (Ye *et al.*, 1985 ▸; Yang *et al.*, 2018 ▸). A large number of star motifs consisting of five fat rhombic tiles can be observed, while a small number of flattened hexagonal tiles are also grouped in fives, forming star motifs in the Zn–Mg–Y quasicrystal. Short ‘zigzag’ packing of several fat rhombic tiles is present randomly in the Zn–Mg–Y quasicrystal, in addition to the star motifs, as shown in Figs. 3 ▸(*b*) and 3(*c*), which is similar to Mg–Zn nanoprecipitates (Yang *et al.*, 2018 ▸). In addition to fat rhombic and flattened hexagonal tiles, one boat tile can be observed, as indicated by the green square in Fig. 3 ▸(*c*). The atomic structure model [Fig. 3 ▸(*d*)] of the boat tile is proposed, based on atomic resolution HAADF-STEM imaging [Fig. 3 ▸(*b*)] and atomic resolution EDX measurements (see Fig. 4 ▸). The above-mentioned boat tile and two flattened hexagonal tiles form a decagon, as shown in Figs. 3 ▸(*b*) and 3(*c*). The boat tile can be decomposed into three fat rhombic tiles and one skinny rhombic tile, as shown schematically in Fig. 3 ▸(*d*). The vertex connecting four edges indicated by dashed lines inside the boat tile should correspond to a column of Zn atoms at *z* = *c*/4, since it is a part of the icosahedral chain centered at the vertex indicated by an arrow at the boundary. This is consistent with the fact that the brightness of the corresponding intensity spot is similar to those at the mid-edge positions, as seen from Fig. 3 ▸(*b*). Given the low number density of the boat tiles, it can be regarded as a kind of defect in this Zn–Mg–Y decagonal quasicrystal.

There are a high fraction of decagonal units consisting of one boat tile and two flattened hexagonal tiles in the proposed model for the Zn–Mg–Dy decagonal quasicrystal (Ors *et al.*, 2014 ▸). In addition, atomic resolution electron microscopy imaging and X-ray diffraction indicated the presence of perfect decagonal units (1.5 nm in diameter) with Dy atoms at the center in the Zn–Mg–Dy decagonal quasicrystal (Ors *et al.*, 2014 ▸). No such perfect decagonal units present in the Zn–Mg–Dy decagonal quasicrystal were observed in the present Zn–Mg–Y decagonal quasicrystal, as shown in Fig. 3 ▸. This indicates that the atomic structure of the present Zn–Mg–Y decagonal quasicrystal is different from the Zn–Mg–Dy decagonal quasicrystal reported by Örs *et al.*, although both crystallize in the same space group *P*10/*mmm* (Ors *et al.*, 2014 ▸). In addition, all fat rhombic tiles were arranged in groups of five, forming star motifs in the model predicted by theoretical calculations for the Zn–Mg–Y decagonal crystal (Mihalkovič *et al.*, 2014 ▸). However, our atomic resolution HAADF-STEM observations showed that almost 25% of fat rhombic tiles were arranged in zigzag motifs in the present Zn–Mg–Y decagonal quasicrystal (Fig. 3 ▸), which differs from the theoretical prediction of star motifs for all fat rhombic tiles (Mihalkovič *et al.*, 2014 ▸). The present Zn–Mg–Y decagonal quasicrystal crystallizes in the space group *P*10/*mmm*, which is different from *P*10_5_/*mmc* for the Zn–Mg–Dy quasicrystal (Abe & Sato, 1998 ▸; Abe *et al.*, 1999 ▸). Therefore, our results demonstrate that another kind of F-K quasicrystal was formed by the Zn–Mg–Y system, in addition to previously reported icosahedral quasicrystals and the decagonal quasicrystal with overlap tiling of quasi-unit-cells or the two-cluster model of decagonal and star-like clusters in Zn-based alloys (Abe & Sato, 1998 ▸; Abe *et al.*, 1999 ▸; Luo *et al.*, 1993 ▸; Liu *et al.*, 2015*a* ▸,*b* ▸; Ors *et al.*, 2014 ▸).

In the case of Mg–Zn Laves phases, atomic columns within fat rhombic and flattened hexagonal units consist purely of Mg atoms; therefore, they could not produce image spots showing intensity high enough in the HAADF-STEM image, due to the low atomic number of Mg (Yang *et al.*, 2018 ▸; Zhang *et al.*, 2019 ▸). Interestingly, an intensity spot can be observed at one of the two sites which is expected to be occupied by Mg in Laves Mg–Zn crystals in some of the fat rhombic tiles, indicating the occurrence of substitution of heavy elements for Mg at such lattice sites. And they are usually at the centers of ‘dented decagon’ motifs composed of three fat rhombic and two flattened hexagonal tiles, as shown in Fig. 3 ▸(*b*). Atomic resolution EDX mapping was carried out in order to obtain chemical information about the substitution. Fig. 4 ▸ shows elemental mapping results for Zn and Y for the region indicated by the red square in Fig. 3 ▸(*b*). It is clear that there are Zn atoms located at the vertices and the middle points of the tile edges, which is the same as in Mg–Zn Laves crystals. Atomic resolution elemental mapping results indicate substitution of Y for Mg at the centers of ‘dented decagon’ motifs, as shown in Fig. 4 ▸(*c*). Interestingly, Y atoms preferentially form the pentagon and skinny rhombus which are the constituents of pentagonal Penrose tiling (Niizeki, 1989 ▸), as indicated by the red lines in Fig. 4 ▸(*b*). The distribution of Mg is not shown in Fig. 4 ▸, since EDX signals of Mg are not high enough for the present Zn–Mg–Y quasicrystal. Based on the atomic resolution HAADF-STEM imaging and EDX measurements, the stoichiometry of the Zn–Mg–Y decagonal quasicrystal is estimated to be Zn_68.3_Mg_29.1_Y_2.6_ (at.%).

**Figure 5:**
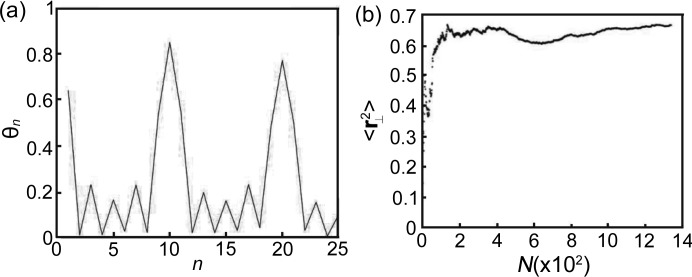
(*a*) Orientational order parameter and (*b*) calculated result of 〈**r**_⊥_^2^〉 of the tiling shown in Fig. 3 ▸(*c*).

To examine the orientation symmetry in local areas of this decagonal quasicrystal, we computed the orientation order parameter, defined as
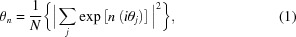
where θ_*j*_ is defined as the angle the *j*th edge of the tiles makes with an arbitrary edge, the sum is over all edges, and *N* is the number of edges (Strandburg, 1989 ▸). For a structure with decagonal symmetry, θ_10_ is expected to be large and θ_*n*_ for *n* (not a multiple of 10) is expected to be small (Strandburg, 1989 ▸). The calculated result is shown in Fig. 5 ▸(*a*), and the existence of tenfold symmetry in the local area of this decagonal quasicrystal is strongly supported. A perfect Penrose tiling cannot be formed by only these two tiles observed in the present Zn–Mg–Y decagonal quasicrystal (Lück, 1990 ▸). This Zn–Mg–Y decagonal quasicrystal may be modeled by phason-perturbed Penrose tiling or random tiling which is stabilized by entropy (Socolar *et al.*, 1986 ▸; Strandburg, 1989 ▸). To clarify this tiling, calculation in hyperspace is performed. Any vertex in an arbitrary tiling of Penrose tiles can be represented as a lattice point in a 5D hyperspace lattice. The procedure for defining the 5D coordinates of a vertex is as follows: (i) choosing an arbitrary vertex as the origin; (ii) ‘walking’ from the origin to the vertex along the edges of the rhombi or flattened hexagons; (iii) counting the number of positive steps minus the negative steps taken along each of the five pentagonal directions. The numbers are assigned to the corresponding coordinates {*n*_*i*_}, where *i* = 1, 2,…, 5. The 5D space can be divided into *E* ^5^ = *E* ^∥^ + *E* ^⊥^+ Δ, where Δ = (1, 1, 1, 1, 1) is the diagonal of the 5D unit cell (Duneau & Katz, 1985 ▸). Every vertex position **r**_∥_ in the *E* ^∥^ physical space can be as a sum of basic vectors with these integer coefficients:
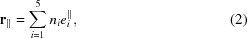
where the basic vectors are 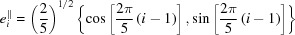
(*i* = 1, 2,…, 5), as shown by the red arrows in Fig. 3 ▸(*c*). The corresponding vertex position **r**_⊥_ in the *E* ^⊥^ perpendicular space spanned by 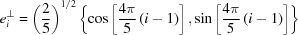
is
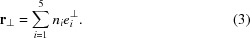
According to the random tiling theories, the mean square deviation of **r**_⊥_ as a function of the number of vertices *N*, 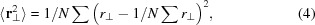
should have a logarithmic increase (Widom *et al.*, 1989 ▸, Strandburg *et al.*, 1989 ▸). However, our 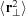 experimental values tend to be convergent when *N* is large enough, as shown in Fig. 5 ▸(*b*). This suggests that the tiling in the present Zn–Mg–Y quasicrystal should be interpreted as a phason-perturbed Penrose tiling, instead of a random tiling (Socolar *et al.*, 1986 ▸).

**Figure 6:**
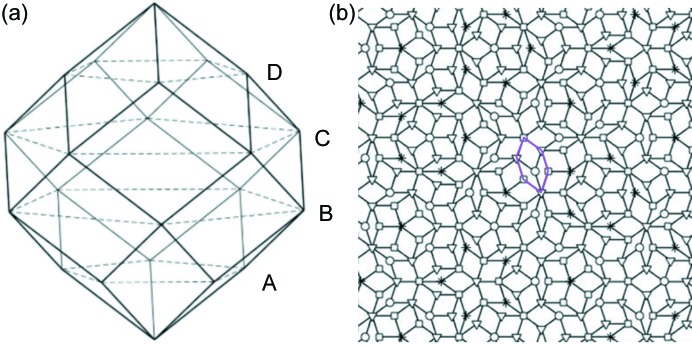
(*a*) Occupation domains in 4D space and (*b*) perfect Penrose tiling.

A perfect Penrose tiling is generated by the four-dimensional section method from four pentagonal occupation domains designated A, B, C and D in Fig. 6 ▸(*a*) (Yamamoto & Ishihara, 1988 ▸; Ishihara & Yamamoto, 1988 ▸). In this method, the Penrose tiling can be decomposed into four sub-lattices [Fig. 6 ▸(*b*)], and the vertices in each sub-lattice (denoted by circles, triangles, squares and asterisks, respectively) are generated by one of the four pentagonal occupation domains. Numerous fat rhombic and skinny rhombic tiles can be seen in the perfect Penrose tiling. However, a very short distance of the short diagonal (*a*/τ < *a*) of skinny rhombic tiles will induce overlap of two icosahedrons, which has not been observed in the F-K compounds. Triangular face sharing of icosahedrons in fat rhombi and flattened hexagons is observed frequently in the Mg–Zn Laves phase (Frank & Kasper, 1958 ▸; Wang *et al.*, 1986 ▸; Ye *et al.*, 1985 ▸; Kuo *et al.*, 1986 ▸; Yang *et al.*, 2018 ▸, Zhang *et al.*, 2018 ▸). In addition, calculations also indicate that fat rhombic and flattened hexagonal tiles with icosahedrons located at vertices have energies lower than other arrangements of icosahedrons (Mihalkovič *et al.*, 2014 ▸). Considering only tilings where the vertices form a subset of vertices of a tiling of the two Penrose rhombi, the highest density is achieved when all skinny rhombi in the parent tiling are paired and form flattened hexagonal tiles [denoted by purple lines in Fig. 6 ▸(*b*)] (Mihalkovič, 1995 ▸; Cockayne, 1995 ▸). This is essentially the same as that of packing equal disks on Penrose tile vertices (Mihalkovič, 1995 ▸; Cockayne, 1995 ▸). Those disks correspond to the icosahedrons in the present model. However, local presence of a skinny rhombic tile was observed in the Zn–Mg–Y decagonal quasicrystal, as shown in Fig. 3 ▸(*d*), suggesting that the present Zn–Mg–Y decagonal quasicrystal can be modeled with an intermediate tiling located between the ideal Penrose tiling and random tiling decorated with icosahedral units at each vertex. This kind of tiling is usually referred to as a phason-perturbed Penrose tiling (Socolar *et al.*, 1986 ▸).

Fat rhombic and/or flattened hexagonal tiles are arranged periodically in binary Mg–Zn compound crystals (*e.g.* MgZn_2_ and Mg_4_Zn_7_), although local domains showing fivefold rotation were observed in some tiny precipitates in an Mg–Zn alloy (Yang *et al.*, 2018 ▸). It has been confirmed that most of the stable quasicrystals are strict electron compounds which only form at sharp valence electron concentration (*e*/*a*) (Tsai, 2004 ▸). The *e*/*a* value of Zn-based quasicrystals is around 2.0–2.15, such as 2.1 for stable icosahedral quasicrystal Zn_6_Mg_3_Y and 2.02 for decagonal quasicrystal Zn_58_Mg_40_Dy_2_ (Tsai, 2004 ▸). In the present study, the stoichiometry of the Zn–Mg–Y decagonal quasicrystal is estimated to be Zn_68.3_Mg_29.1_Y_2.6_ (at.%) with an *e*/*a* value of about 2.03 which is quite close to that for the Zn_58_Mg_40_Dy_2_ decagonal quasicrystal. A previous study showed that an orthorhombic MgYZn_4_ phase (space group *Pmnn*, *a* = 0.53, *b* = 0.95, *c* = 0.86 nm) forms if 50% of Mg in MgZn_2_ are substituted by Y (Zhang *et al.*, 2019 ▸). The value of *e*/*a* for MgYZn_4_ is 2.17, which is above the upper limit of 2.15 for the formation of Zn–Mg–RE quasicrystals (Tsai, 2004 ▸). Therefore, the presence of a suitable concentration of trivalent RE elements (*e.g.* Y in the present study) plays an important role in the formation of Zn–Mg–RE quasicrystals.

*In situ* high-temperature X-ray diffraction experiments indicated that the Zn–Mg–Dy decagonal quasicrystal showed the best quasiperiodic order (lowest phasonic disorder) at 598 K, and increasing the temperature to 648 K would worsen the quasiperiodic ordering (Ors *et al.*, 2014 ▸). Interestingly, no obvious changes in atomic structures were observed in the present Zn–Mg–Y decagonal quasicrystal subjected to annealing at 673 K for 100 h, implying that the present Zn–Mg–Y decagonal quasicrystal had fairly good thermodynamic stability. *Ab initio* calculations found that: (i) the binary Mg–Zn decagonal quasicrystal was unstable by 1.1 meV per atom, using an artificial approximant model based on a supercell (with 56 atoms) consisting of two hexagons and one star motif; (ii) the substitution of a Y atom for one Mg atom requires an energy cost of ∼90 meV; (iii) the Zn–Mg–Y decagonal quasicrystal becomes thermodynamically stable when the temperature exceeds 705 K (Mihalkovič *et al.*, 2014 ▸). The theoretical calculations indicated that substitutional entropy could compensate the energy penalty and destabilize the Zn–Mg–Y decagonal quasicrystal, which consequently plays an important role in stabilizing the Zn–Mg–Y decagonal quasicrystal at higher temperatures (Mihalkovič *et al.*, 2014 ▸). Our experimental results showed the formation of one Zn–Mg–Y decagonal quasicrystal with an intermediate structure lying between the ideal Penrose tiling and random tiling of fat rhombic and flattened hexagonal tiles decorated with icosahedral units at each vertex. The Zn–Mg–Y decagonal quasicrystal could be stable at temperatures far below 705 K, and even at ambient temperature. In addition, it was pointed out that the stabilization of the Zn–Mg–Y decagonal quasicrystal should be assisted by the fact that the quasicrystal had a variety of local environments including Mg sites that are favorable for Y (Mihalkovič *et al.*, 2014 ▸). But interestingly, substitution of Y for Mg was observed only at the center of the ‘dented decagon’ motif in the present Zn–Mg–Y decagonal quasicrystal, as shown in Figs. 3 ▸ and 4 ▸. This suggests that the energy penalty plays a critical role in site occupation for Y substitutions, since the energy cost was calculated to be 210–270 meV in other sites of the outer ring of the star motif in the approximant model, while it was about 90 meV for the ‘dented decagon’ center (Mihalkovič *et al.*, 2014 ▸).

## 4. Conclusions   

A decagonal quasicrystal with periodicity 0.51 nm along the tenfold axis and space group *P*10/*mmm* is discovered in a Zn_58_Mg_40_Y_2_ (at.%) alloy. The composition of this decagonal quasicrystal is estimated to be Zn_68.3_Mg_29.1_Y_2.6_ (at.%). Atomic resolution HAADF-STEM observations reveal that the structure of this decagonal quasicrystal can be modeled with a rhombic/hexagonal tiling decorated with icosahedral units at each vertex. The structure of the present Zn–Mg–Y decagonal quasicrystal is not only different from that of the Zn–Mg–Dy decagonal quasicrystal, but also different from the theoretically predicted structure for the Zn–Mg–Y decagonal quasicrystal in terms of the arrangement of fat rhombic tiles. This decagonal quasicrystal composed of simple icosahedral units may be a good candidate to investigate the formation and growth of quasicrystals theoretically and experimentally.
